# Real-World Outcomes for Patients with Clinically Node-Positive Melanoma Undergoing Neoadjuvant Immunotherapy and Nodal Dissection

**DOI:** 10.1245/s10434-026-19130-1

**Published:** 2026-03-16

**Authors:** Joshua Herb, Roland L. Bassett, Carlos A. Torres-Cabala, Victor G. Prieto, Sapna P. Patel, Ashley M. Holder, Sarah B. Fisher, Anthony Lucci, Jennifer Wargo, Rodabe N. Amaria, Michael A. Davies, Isabella C. Glitza Oliva, Hussein A. Tawbi, Jennifer McQuade, Alexandra P. Ikeguchi, Michael K. Wong, Adi K. Diab, Jeffrey E. Gershenwald, Merrick I. Ross, Roi Weiser

**Affiliations:** 1 Department of Surgical Oncology, The University of Texas MD Anderson Cancer Center, Houston, TX USA; 2 Department of Biostatistics, The University of Texas MD Anderson Cancer Center, Houston, TX USA; 3 Department of Pathology, The University of Texas MD Anderson Cancer Center, Houston, TX USA; 4 Department of Medicine, Division of Medical Oncology, University of Colorado, Boulder, CO USA; 5 Department of Melanoma Medical Oncology, The University of Texas MD Anderson Cancer Center, Houston, TX USA; 6 Department of Medicine, Division of Medical Oncology, Roswell Park Comprehensive Cancer Center, Buffalo, NY USA

## Abstract

**Background:**

Neoadjuvant immunotherapy (nIO) followed by a therapeutic lymph node dissection (TLND) for patients with clinically node-positive melanoma is supported by level 1 evidence. Our aim was to assess the association of pathologic response (PR) and nIO regimen with survival among patients with clinical stage III melanoma undergoing nIO and TLND.

**Methods:**

Our study included patients treated with nIO followed by a TLND from a large academic institution between 2016 and 2024. The pathologic response was assessed using established guidelines. Differences in extent of PR by nIO regimen were examined. Disease-free survival (DFS) and overall survival (OS) by PR and nIO regimen were assessed.

**Results:**

The study included 121 patients (78.5% treated with combination nIO; 21.5% treated with single-agent anti-PD-1). The pCR rate was 64.2% for combination therapy and 53.8% for monotherapy (*P*=0.11). In univariable analysis, patients who received combination therapy had a 2-year DFS similar to patients treated with single-agent anti-PD-1 (86.5% vs 85.1%, respectively;* P*=0.43). Patients with a pCR had a 2-year DFS of 94.4% versus 57.3% among those with pathologic non-response (pNR) (*P*<0.001). In multivariable analysis, PR was associated with DFS, but the nIO regimen was not. Our findings showed similar results for OS, with a 2-year OS of 98.1% for patients with pCR compared with 94.7% for those with pNR (*P*=0.01).

**Conclusions:**

Consistent with clinical trial data, the findings in this study support PR as a strong predictor of survival in this patient population. Studies investigating the de-escalation of surgical and systemic treatment based on PR as well as biomarkers of patients who can achieve a pCR after monotherapy alone are warranted.

**Supplementary Information:**

The online version contains supplementary material available at 10.1245/s10434-026-19130-1.

Immunotherapy (IO) has revolutionized the treatment of patients with advanced melanoma. Patients with resectable stage III or IV disease have been shown to derive recurrence-free survival (RFS) benefit from adjuvant systemic single-agent programmed cell death protein-1 (PD-1) inhibitors after surgery.^1–3^ Small-cohort studies evaluating IO in the neoadjuvant setting in a similar patient population showed feasibility, safety, and impressive response rates.^4,5^

Recently, the SWOG S1801 and NADINA trials further showed superiority in outcomes when IO (pembrolizumab in SWOG S1801; combination ipilimumab and nivolumab in NADINA) was used in the neoadjuvant and potentially adjuvant setting compared with the adjuvant setting alone.^6,7^ As a result, National Comprehensive Cancer Network (NCCN) guidelines recommend neoadjuvant IO (nIO) followed by therapeutic lymph node dissection (TLND) for clinically node-positive stage III patients as the standard of care.^8^

The neoadjuvant setting helps differentiate patients based on their response to treatment, providing valuable prognostic information.^6,7,9,10^ Patients treated with nIO who achieve a pathologic complete response (pCR) or near-pCR, and to some extent even a partial response (pPR), have been shown to have durable responses and a considerably better RFS than patients with a pathologic non-response (pNR) to nIO.

These findings help stratify patients based on prognosis and could inform subsequent surgical management and adjuvant treatment. For example, the NADINA study tailored adjuvant IO based on pathologic response (PR) in the resected specimens,^7^ and the PRADO trial explored tailoring the extent of lymphatic surgery as well as adjuvant IO based on the extent of PR in an “index” node.^9^

Real-world evidence can often complement data from randomized controlled trials.^11^ Such data in melanoma patients who receive nIO and TLND could validate recent level 1 evidence. Additionally, there are limited data directly comparing monotherapy versus combination immunotherapy in the neoadjuvant setting for stage III melanoma.^4,5^ Thus, the objective of our study was to evaluate response rates, disease-free survival (DFS), and overall survival (OS) for patients with clinically node-positive stage III melanoma who received neoadjuvant monotherapy or combination nIO at a large academic cancer center.

## Methods

### Study Design

This retrospective cohort study analyzed patients treated at the University of Texas MD Anderson Cancer Center. Patients with a diagnosis of clinically detected and pathologically confirmed node-positive melanoma undergoing curative-intent nIO followed by TLND between 2016 and 2024 were identified from the electronic medical records (Supplementary Appendix and Fig. S1). Because we aimed to assess pathologic response, the study included only patients who underwent TLND. Both patients with de novo clinical stage III disease and those with nodal recurrence were included. The study excluded patients with mucosal melanoma and those who received prior IO, targeted therapy, or intralesional therapy. The study further excluded patients who had incomplete pathologic evaluation (patients who did not have a quantified percentage of viable tumor remaining). Patients with distant metastatic disease and those treated with non–standard-of-care regimens during their neoadjuvant course also were excluded. Patients participating in nIO clinical trials investigating regimens currently used in practice were not excluded.

Patient demographics and disease history, including primary tumor characteristics and type, if any, of prior locoregional therapy, were recorded. Neoadjuvant regimen, number of cycles, site of involved nodal basin, and adjuvant treatments were assessed. Adjuvant treatment (systemic or radiation) was based on physician and patient preference or trial protocol. Follow-up time until date of recurrence, date of last follow-up visit, and/or date of death also were recorded.

The primary study variable was the neoadjuvant regimen, defined as monotherapy (single-agent anti-PD-1 [either pembrolizumab or nivolumab]) or combination therapy. Combination regimens included anti-CTLA4 (ipilimumab) and nivolumab (either in the standard 3 mg/kg ipilimumab and 1 mg/kg nivolumab dose or the “flip-dose” 1 mg/kg ipilimumab and 3 mg/kg nivolumab), or nivolumab and anti-LAG-3 (relatlimab).

The primary study outcomes were PR and DFS, with OS included as a secondary outcome. Pathologic assessment of lymph node basin specimens was performed by dedicated dermatopathologists, and response to neoadjuvant therapy was scored according to the International Neoadjuvant Melanoma Consortium (INMC)^12^ guidelines and categorized as complete pathologic response (pCR: 0% viable tumor), near-complete pathologic response (near-pCR: 1–10% viable tumor), partial pathologic response (pPR: 11–50% viable tumor), or pathologic non-response (pNR: 51–100% viable tumor). DFS was calculated from time of surgery until recurrence, death, or date of last follow-up visit. OS was calculated from time of surgery until death or date of last follow-up visit.

### Statistical Analysis

Baseline characteristics were compared across neoadjuvant regimens using Fisher’s exact test for categorical variables and Wilcoxon rank-sum tests for continuous variables. Kaplan-Meier analysis was used to estimate the distribution of DFS and OS by PR, and log-rank tests were used to compare survival distributions. Similar methods were used to compare survival parameters by neoadjuvant regimen. Uni- and multivariable Cox regression analyses were used to assess factors associated with DFS. Due to a low number of DFS events, a limited number of variables were included in the adjusted multivariable model, based on the univariable survival analysis. These variables included PR, neoadjuvant regimen, initial presentation versus recurrence, number of involved lymph nodes, and presence of satellite or in-transit disease. Due to a low number of patients with near-pCR, these patients were combined in the regression analysis with the pCR group into a “major pathologic response” (MPR) group.

All *p* values were two-sided and used a significance level of 0.05. The study was approved by the Institutional Review Board (IRB) at The University of Texas MD Anderson Cancer Center (IRB protocol no. 2023-0971).

## Results

### Baseline Characteristics

The study included 121 patients. Clinicopathologic data are summarized in Table 1. The median age was 61.2 years (interquartile range [IQR], 50.7–70.6 years). The majority of the cohort was male (75.2%), and most had an Eastern Cooperative Oncology Group performance status (ECOG PS) of 0 (75.2%). These data did not differ statistically between those receiving monotherapy and those receiving combination therapy. Of the 121 patients, 28 (23.1%) had melanoma of unknown primary metastatic to lymph nodes. Of the 93 patients (76.9%) with known cutaneous primary tumors, 7 (5.8%) had acral tumors. For 87 patients with fully evaluable primary tumors, the median Breslow thickness was 2.3 mm (IQR, 1.2–4.1 mm), 37.5% of the tumors were ulcerated, and the median number of mitoses was 7 (IQR, 3–12.5). These factors did not differ between the monotherapy and combination therapy groups.

**Table 1:** Demographic and clinical characteristics

Characteristic	Total (*n* = 121) *n* (%)	Monotherapy (*n* = 26) *n* (%)	Combination therapy (*n* = 95) *n* (%)	*P* value
Median age: years (IQR)	61.2 (50.7–70.6)	69.3 (58.0–79.2)	60.1 (49.7–67.2)	0.003
Male sex	91 (75.2)	19 (73.1)	72 (75.8)	0.80
ECOG PS				0.36
0	91 (75.2)	17 (65.4)	74 (77.9)	
1–2	30 (24.8)	9 (34.6)	21 (22.1)	
Pattern of presentation				0.062
Primary tumor and nodal disease	43 (35.5)	9 (34.6)	34 (35.8)	
Nodal disease and unknown primary	28 (23.1)	2 (7.7)	26 (27.4)	
Nodal disease recurrence with primary resected in the past	50 (41.3)	15 (57.7)	35 (36.8)	
SLNB not performed at time of past primary excision	16 (32.0)	5 (33.3)	11 (31.4)	0.52
SLNB performed at time of past primary excision	34 (68.0)	10 (66.7)	24 (68.6)	0.26
Positive	11 (32.4)	5 (50.0)	6 (25.0)	
Negative	23 (67.6)	5 (50.0)	18 (75.0)	
Primary tumor type				0.07
Cutaneous	93 (76.9)	24 (92.3)	69 (72.6)	
Acral	7 (5.8)	1 (3.8)	6 (6.3)	
Unknown primary	28 (23.1)	2 (7.7)	26 (27.4)	
Median Breslow thickness: mm (IQR; *n* = 87)	2.3 (1.2–4.1)	1.85 (0.9–4.1)	2.4 (1.3–3.1)	0.27
Ulceration (*n* = 80)	30 (37.5)	8 (36.4)	22 (37.9)	1.00
Median mitotic rate (IQR; *n* = 80)	7 (3–12.5)	8 (2.25–14.75)	7 (3–12)	0.75
Nodal basin location				0.08
Axillary	42 (34.7)	6 (23.1)	36 (37.9)	
Groin	21 (17.4)	2 (7.7)	19 (20.0)	
Head & neck	53 (43.8)	17 (65.4)	36 (37.9)	
Multiple	5 (4.1)	1 (3.8)	4 (4.2)	
Satellite or in-transit disease present at time of TLND	8 (6.6)	4 (15.4)	4 (4.2)	0.06
Median no. of involved lymph nodes in specimen (IQR)	1 (1–3)	2 (1–3)	1 (1–3)	0.61
BRAF mutant (*n* = 111)	38 (34.2)	6 (28.6)	32 (35.6)	0.62

*IQR* Interquartile range, *ECOG PS* Eastern Cooperative Oncology Group performance status, *SLNB* Sentinel lymph node biopsy, *TLND* Therapeutic lymph node dissection, *BRAF* v-raf murine sarcoma viral oncogene homolog B1

Overall, 71 (58.7%) of the 121 patients had clinically node-positive disease as their initial presentation (with 35.5% having an intact primary and 23.1% having an unknown primary), whereas the remaining 50 patients (41.3%) experienced nodal recurrence after prior primary tumor surgery. Of these 50 patients, 34 (68.0%) had undergone a sentinel lymph node biopsy (SLNB) in the past and had SLNB data available, 11 (32.4%) had a positive SLNB, and 8 patients had undergone a prior TLND.

The most frequently involved nodal basin at the time of nIO was head and neck (43.8%), followed by axillary (34.7%), groin (17.4%), and multiple nodal basins (4.1 %). Patients in the monotherapy group more frequently had involved head and neck nodal basins (65.4% vs 37.9%) compared to patients in the combination therapy group, and less frequently had involved axillary nodes (23.1% vs 37.9%), but this difference was not statistically significant (*P*=0.08).

### Treatment Characteristics

Patient treatment characteristics are summarized in Table 2. Most of the patients (*n*=95, 78.5%) received combination nIO with either ipilimumab/nivolumab (*n*=73, 60.3%) or nivolumab/relatlimab (*n*=22, 18.2%), whereas 21.5% (*n*=26) received monotherapy. For 30 patients (24.8%), nIO was received as part of a clinical trial. The median number of neoadjuvant cycles was 2 (IQR, 2–3). The median number of lymph nodes removed at the time of lymph node dissection was 28 (IQR, 18–38), and the median number of pathologically involved nodes (including those with a pCR) was 1 (IQR, 1–3). On final pathology, 65 (53.7%) of the patients had only one involved node, 22 (18.2%) had two involved nodes, 8 (6.6%) had three involved lymph nodes, 5 (4.1%) had four involved lymph nodes, and 21 (17.4%) had five or more involved nodes.

**Table 2:** Treatment characteristics

Characteristic	Total (*n* = 121) *n* (%)
Neoadjuvant regimen	
Combination therapy	95 (78.5)
Ipilimumab/nivolumab	73 (60.3)
Nivolumab/relatlimab	22 (18.2)
Monotherapy (pembrolizumab or nivolumab)	26 (21.5)
Median no. of neoadjuvant cycles (IQR)	2 (2–3)
Median no. of total lymph nodes removed (IQR)	28 (18–38)
Median no. of pathologically involved lymph nodes in TLND specimen after nIO (IQR)	1 (1–3)
No. of patients with 1 patholigically involved lymph node in TLND specimen after nIO	65 (53.7)
Adjuvant systemic therapy	95 (78.5)
Immunotherapy	89 (73.6)
Targeted therapy	6 (5.0)
Adjuvant radiation to nodal basin	17 (14.0)

*IQR* Interquartile range, *TLND* Therapeutic lymph node dissection, *nIO* Neoadjuvant immunotherapy

Overall, 78.5% of the patients received adjuvant systemic therapy after TLND, based on patient and physician preference, with 73.6% receiving adjuvant immunotherapy and 5.0% receiving targeted therapy. Among those who received adjuvant systemic therapy after TLND, 59 (62.1%) had a pCR, 4 (4.2%) had near-pCR, 14 (14.7%) had pPR, and 18 (18.9%) had pNR in their specimens. The percentage of patients who received adjuvant systemic treatment after TLND was similar for each pathologic response group: 78.7% of the pCR group, 80.0% of the near-pCR group, 77.8% of the pPR group, and 78.3% of the pNR group. A minority of the patients (14%) received adjuvant nodal basin radiation.

### Response Rates

**Fig. 1:**
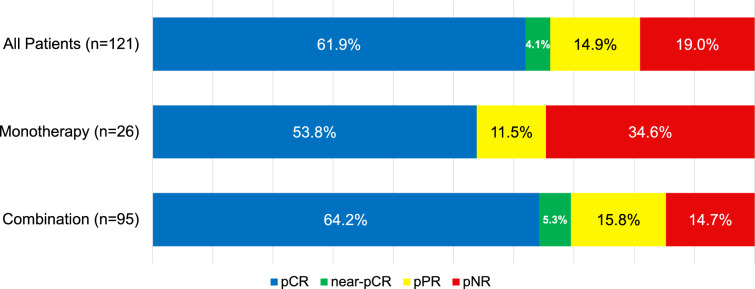
Pathologic response rates by neoadjuvant immunotherapy regimen. Fisher’s exact test compares monotherapy and combination therapy (*P*=0.11)

The overall PR rates for the entire cohort stratified by nIO regimen are shown in Fig. 1. Among all the patients, 61.9% (*n*=75) of the TLND specimens demonstrated pCR, 4.1% (*n*=5) demonstrated near-pCR, 14.9% (*n*=18) demonstrated pPR, and 19.0% (*n*=23) demonstrated pNR. In the monotherapy group, 53.8% had a pCR and 34.6% had a pNR, compared with the combination therapy group in which 64.2% had a pCR and 14.7% had a pNR. However, these differences did not reach statistical significance (*P*=0.11, Fisher exact test).

### Disease-Free Survival

The median follow-up period was 23 months. During this period, 17 patients experienced recurrence. The primary site of recurrence was locoregional in 4 patients (1 local, 2 in-transit, and 1 in the dissected nodal basin) and distant in 13 patients (4 M1a, 4 M1b, 1 M1c, and 4 M1d). The 2-year DFS for the entire cohort was 85.9%, and the median DFS was not reached.

**Fig. 2:**
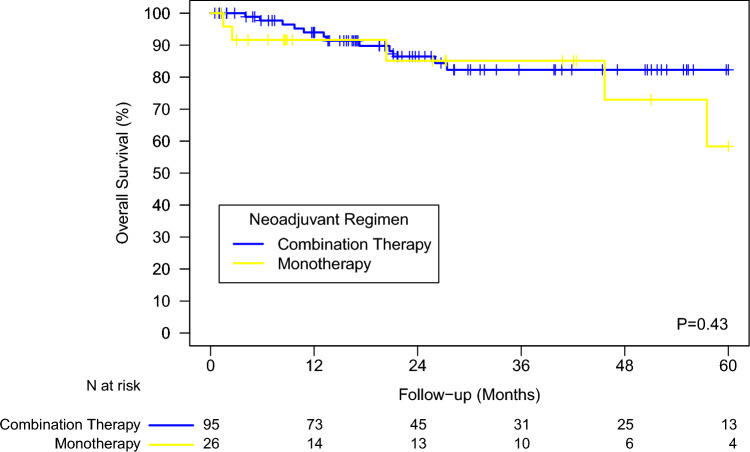
Overall survival by neoadjuvant immunotherapy regimen

In the univariate analysis of DFS by nIO regimen, the 2-year DFS was 86.5% for the patients receiving combination therapy compared with 85.1% for those receiving monotherapy (*P*=0.43, log-rank; Fig. 2).

**Fig. 3:**
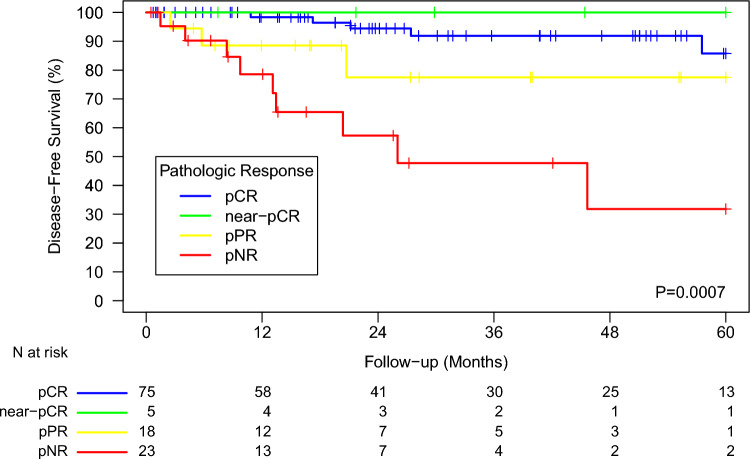
Disease-free survival by extent of pathologic response to neoadjuvant mmunotherapy

DFS differed by extent of PR (Fig. 3). The patients achieving pCR had a 2-year DFS of 94.4%, whereas the 2-year DFS was 100% for those with near-pCR, 77.5% for those with pPR, and 57.3% for those with pNR. These differences in DFS were statistically significant, with an overall log-rank P lower than 0.001.

In multivariable Cox-proportional hazards modeling including our main study variables of PR and nIO regimen, we found that pNR was independently associated with worse DFS compared to MPR (adjusted hazard ratio [aHR], 11.98; 95% confidence interval [CI], 3.34–43.00; overall *P*<0.001). When the analysis accounted for PR, it showed no association of nIO regimen (monotherapy vs combination therapy) with DFS (aHR, 0.56; 95% CI, 0.17–1.87; *P*=0.33). The multivariable analysis further showed that DFS was associated with the number of pathologically involved lymph nodes (aHR, 1.15; 95 % CI, 1.03–1.28; *P*=0.01) and the presence of satellites or in-transit disease at initiation of nIO (aHR, 15.38; 95 % CI, 3.26–72.50; *P*=0.003). Initial presentation versus nodal recurrence was not significant in the multivariable analysis (Table 3).

**Table 3:** Uni- and multivariable Cox proportional hazards analysis of factors associated with disease-free survival

Factor	HR (95 % CI)	*P* value	aHR (95 % CI)	*P* value
Pathologic response		< 0.001		< 0.001
MPR	Reference		Reference	
pPR	3.03 (0.75–12.21)		3.64 (0.83–15.92)	
pNR	8.66 (3.05–24.56)		11.98 (3.34–43.00)	
nIO regimen		0.43		0.33
Combination	Reference		Reference	
Monotherapy	1.54 (0.55–4.35)		0.56 (0.17–1.87)	
Age	1.01 (0.97–1.04)	0.76		
Sex		1.00		
Female	Reference			
Male	1.00 (0.33–3.04)			
ECOG PS		0.83		
0	Reference			
1–2	0.89 (0.29–2.68)			
Initial presentation vs recurrence		0.03		0.18
Recurrence	Reference		Reference	
Initial	0.36 (0.14–0.96)		0.48 (0.16–1.45)	
Breslow thickness (*n* = 87)	1.03 (0.84–1.27)	0.76		
Mitoses (*n* = 80)	0.98 (0.94–1.02)	0.29		
Ulceration (*n* = 80)		0.94		
No	Reference			
Yes	0.96 (0.33–2.83)			
BRAF (*n* = 111)		0.06		
Mutant	Reference			
Wild type	0.41 (0.16–1.02)			
Prior positive SLN (*n* = 36)	1.71 (0.28–10.63)	0.56		
No. of involved lymph nodes	1.08 (0.99–1.18)	0.11	1.15 (1.03–1.28)	0.01
Nodal location		0.38		
Axilla	Reference			
Groin	2.22 (0.45–11.02)			
Head & neck	2.62 (0.73–9.39)			
Multiple	4.35 (0.44–42.62)			
Satellite or in-transit disease resected at time of TLND		0.03		0.003
No	Reference		Reference	
Yes	5.33 (1.51–18.83)		15.38 (3.26–72.50)	
Neoadjuvant cycles	1.02 (0.69–1.51)	0.91		
Adjuvant systemic therapy		0.55		
No	Reference			
Yes	0.70 (0.23–2.18)			

*HR* Hazard ratio, *CI* Confidence interval, *aHR* adjusted hazard ratio, *MPR* Major pathologic response (0–10 % viable tumor), *pPR* pathologic partial response, *pNR* pathologic non-response, *nIO* neoadjuvant immunotherapy, *ECOG PS* Eastern Cooperative Ongcology Group performance score, *BRAF* v-raf murine sarcoma viral oncogene homolog B1, *SLN* Sentinel lymph node, *TLND* Therapeutic lymph node dissection

### Overall Survival

The 2-year OS for the entire cohort was 95.7 % (the median OS was not reached). In the univariable analysis, the 2-year OS was 93.3% for those who had monotherapy compared with 96.1% for those who had combination therapy, with this difference not reaching statistical significance (Fig. S2; *P*=0.66, log-rank). Similar to DFS, OS varied according to pathologic response. The patients achieving pCR or near-pCR had 2-year OS rates of 98.1% and 100%, respectively, whereas those with a pPR or pNR had 2-year OS rates of 86.2% and 94.7%, respectively (*P*=0.01, overall log-rank; Fig. S3).

## Discussion

This study represents the largest single-center cohort assessment of patients undergoing nIO followed by TLND for clinically node-positive stage III melanoma. We found that, consistent with phase 2 and 3 prospective studies, pathologic response in the lymph node basin was associated with improved DFS and OS. Combination therapy did not show improved survival over monotherapy in either the uni- or multivariable analysis, suggesting that the pathologic response drives survival rather than nIO regimen per se.

Our study found response and survival rates similar to those in the two largest randomized controlled trials to date. The MPR rate of 53.8% in our monotherapy regimen group was comparable the 51.7% MPR rate seen in the SWOG S1801 central pathologic review.^13^ The rate of MPR in our combination therapy cohort (69.5%) was comparable with the rate seen in NADINA (59.0%).

Our study also provides a unique comparative analysis of nIO combination therapy versus monotherapy, yet to be compared prospectively in this population. We found a 2-year DFS of 86.5% for the patients treated with combination therapy and a DFS of 85.1 % for those treated with monotherapy. These findings are comparable with the 24-month EFS of 77% reported in the NADINA trial for patients treated with combination ipilimumab and nivolumab and the 2-year EFS of 72% for the patients treated with neoadjuvant pembrolizumab in SWOG S1801.^6,14^

Similar to previous analyses, our data demonstrate that survival outcomes are excellent for patients who experience a pCR after nIO, regardless of combination therapy versus monotherapy. We found better DFS and OS for patients with pCR compared to those with pPR or pNR, including a DFS rate of 94.4% for patients with pCR. These findings are similar to the data by Menzies et al.,^4^ who found RFS at 2 years to be excellent among patients with a pCR (96%) compared to those without a pCR (64%).

Our multivariable analysis demonstrated that independent of immunotherapy regimen and other clinical characteristics, PR was a statistically significant factor associated with DFS. pCR rates appear to be higher among patients receiving combination therapy than among those receiving monotherapy, but patients achieving pCR with either regimen seem to derive similar benefit in terms of outcomes. In choosing an nIO regimen (combination therapy vs monotherapy), a higher toxicity profile would be balanced against the potential absolute higher pCR rates (10.4% in our data). Consequently, biomarkers that would identify patients who have a high likelihood of pCR with a less toxic monotherapy regimen versus those who would benefit from combination therapy are needed to guide clinicians in nIO regimen selection.

Future work should also focus on utilizing PR in tailoring both lymphatic surgery and adjuvant systemic therapy. This concept was studied in NADINA wherein the use of adjuvant systemic therapy and in PRADO wherein the use of adjuvant systemic therapy as well as the performance of TLND were dictated by the PR in the lymphatic basin.^7,9^ In PRADO, the response in the lymphatic basin was estimated by the response in an excised index node. Reijers et al.^15^ went further and retrospectively evaluated how well an index node represented the response in the entire nodal basin of patients in the neoadjuvant cohorts of OpACIN and OpACIN-neo, showing 96% representativeness. Although promising, this limited data have yet to bring the concept of an index node resection into common practice. Our data, demonstrating a high rate of pCR after nIO, with more than 50% of patients having only one involved node and a low rate of lymphatic recurrence, support further investigation into the use of an index node resection in de-escalating lymphatic surgery. The upcoming MSLT-III trial will hopefully address this gap in knowledge.

The strength of our study was that it contained a large, heterogeneous cohort of patients with clinically detected stage III melanoma who underwent nIO with standardized pathologic review. However, this study had limitations. First, it was a single-institution retrospective study at a high-volume academic center, which might have introduced selection bias and may limit generalizability.

Second, although the study had a relatively large cohort compared with prior literature, sample size and number of events may have been insufficient for a robust multivariable analysis. Some patients also were treated as part of a clinical trial, which may limit generalizability.

Finally, because we included only patients who had a TLND and did not include patients who progressed with nIO and did not undergo surgery, our reported response rates likely overestimated the response among all patients receiving nIO.

## Conclusions

In a large cohort of patients treated with nIO for clinically detected node-positive stage III melanoma, we found high rates of pCR and DFS that align with randomized prospective trials. Pathologic response was the primary determinant of long-term DFS, independent of neoadjuvant regimen.

## Supplementary Information

Below is the link to the electronic supplementary material.

Supplementary file1 (DOCX 185 kb) Fig. S1 Selection diagram for derivation of study cohort. Fig. S2 Overall survival by neoadjuvant immunotherapy regimen. Fig. S3 Overall survival by pathologic response of patients receiving neoadjuvant immunotherapy
