# Correction: Real-World Outcomes for Patients with Clinically Node-Positive Melanoma Undergoing Neoadjuvant Immunotherapy and Nodal Dissection

**DOI:** 10.1245/s10434-026-19737-4

**Published:** 2026-04-24

**Authors:** Joshua Herb, Roland L. Bassett, Carlos A. Torres-Cabala, Victor G. Prieto, Sapna P. Patel, Ashley M. Holder, Sarah B. Fisher, Anthony Lucci, Jennifer Wargo, Rodabe N. Amaria, Michael A. Davies, Isabella C. Glitza Oliva, Hussein A. Tawbi, Jennifer McQuade, Alexandra P. Ikeguchi, Michael K. Wong, Adi K. Diab, Jeffrey E. Gershenwald, Merrick I. Ross, Roi Weiser

**Affiliations:** 1 Department of Surgical Oncology, The University of Texas MD Anderson Cancer Center, Houston, TX USA; 2 Department of Biostatistics, The University of Texas MD Anderson Cancer Center, Houston, TX USA; 3 Department of Pathology, The University of Texas MD Anderson Cancer Center, Houston, TX USA; 4 Department of Medicine, Division of Medical Oncology, University of Colorado, Boulder, CO USA; 5 Department of Melanoma Medical Oncology, The University of Texas MD Anderson Cancer Center, Houston, TX USA; 6 Department of Medicine, Division of Medical Oncology, Roswell Park Comprehensive Cancer Center, Buffalo, NY USA

**Correction to: Annals of Surgical Oncology** 10.1245/s10434-026-19130-1

In the original online version of this article, there were errors in Fig. 1 and an error in the fifth paragraph of the Discussion section. The correct sentence is as follows:

In choosing an nIO regimen (combination therapy vs monotherapy), a higher toxicity profile would be balanced against the potential absolute higher pCR rates (10.4% in our data).

The correct Fig. 1 is as follows:

**Fig. 1:**
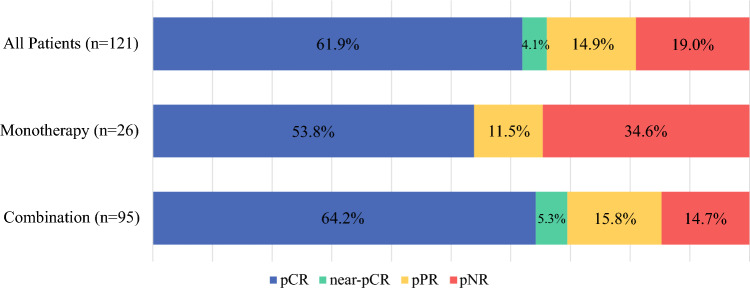
Pathologic response rates by neoadjuvant immunotherapy regimen. Fisher’s exact test compares monotherapy and combination therapy (*P* = 0.11)

The original article was corrected.

