# Supplementary material for: Real-World Outcomes for Patients with Clinically Node-Positive Melanoma Undergoing Neoadjuvant Immunotherapy and Nodal Dissection
Source: Ann Surg Oncol. 2026 Mar 16;33(7):6700–8. doi: 10.1245/s10434-026-19130-1 (PMC13242473; doi:10.1245/s10434-026-19130-1)

**Supplemental Patient Selection Criteria:**

Patients were selected from our electronic medical record (EPIC Systems, Madison Wisconsin) using Current Procedural Terminology codes, and International Classification of Diseases 10^th^ edition codes for melanoma, and further refined to patients undergoing “neoadjuvant therapy” or “induction therapy” as noted in the medical record.

Procedure codes: 38500; 38510; 38570; 38571; 38760; 38780; 38745; 38765; 27337; 38724; 38700; 38720

ICD-10 Codes: C43.10; C43.11; C43.111; C43.112; C43.121; C43.20; C43.21; C43.22; C43.3; C43.5; C43.6; C43.61, C43.62; C43.7; C43.8; C43.9, C78.89; C43.9, C79.2; C43.9, C80.2, T86.99; C43.9, L81.4; C77.9, C43.9; C78.00, C43.9; C79.89, C79.2, C43.9, C77.9; C80.0, C43.9; L81.6, C43.9; C43.9; C43.4; C43.39; C43.62; C43.71; C43.59; C43.51; C43.31; C43.72

**Supplemental Figure 1: Selection diagram for derivation of study cohort**


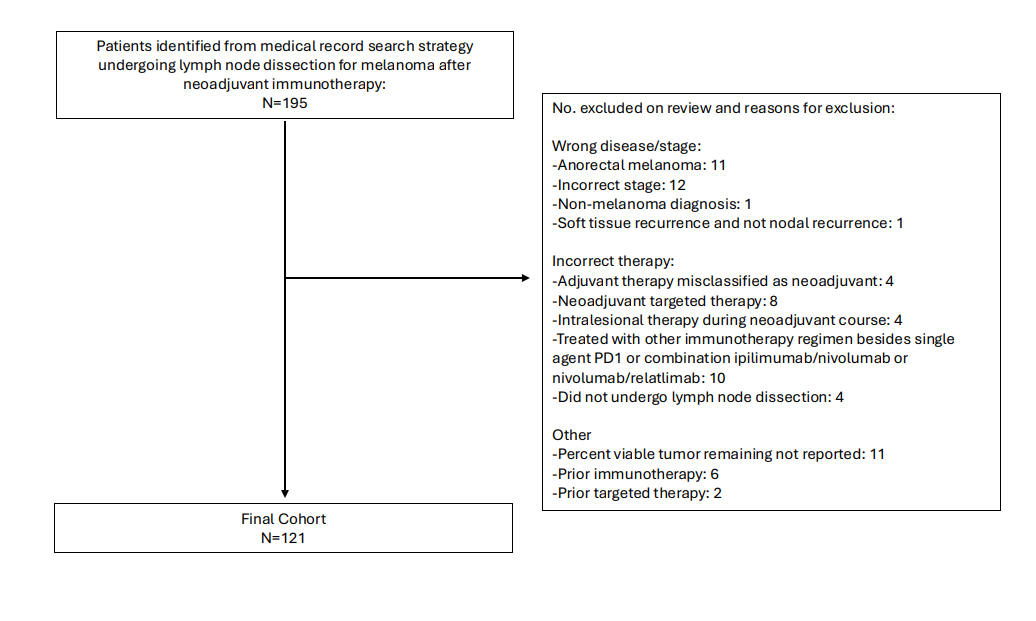


**Supplemental Figure 2: Overall survival by neoadjuvant immunotherapy regimen**


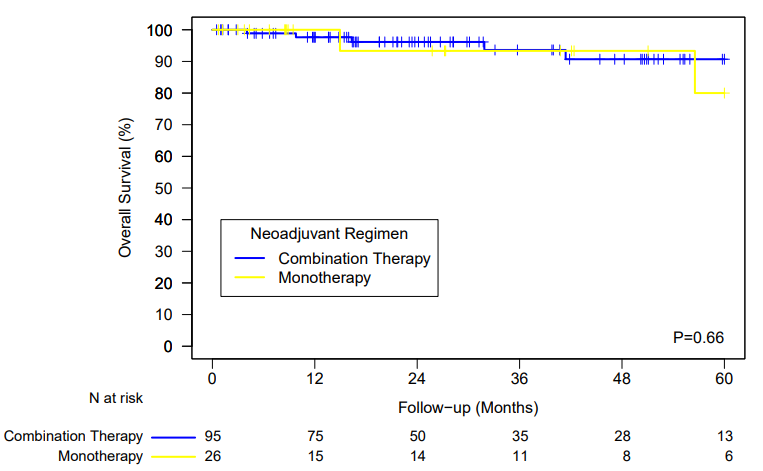


**Supplemental Figure 3: Overall survival by pathologic response in patients receiving neoadjuvant immunotherapy**


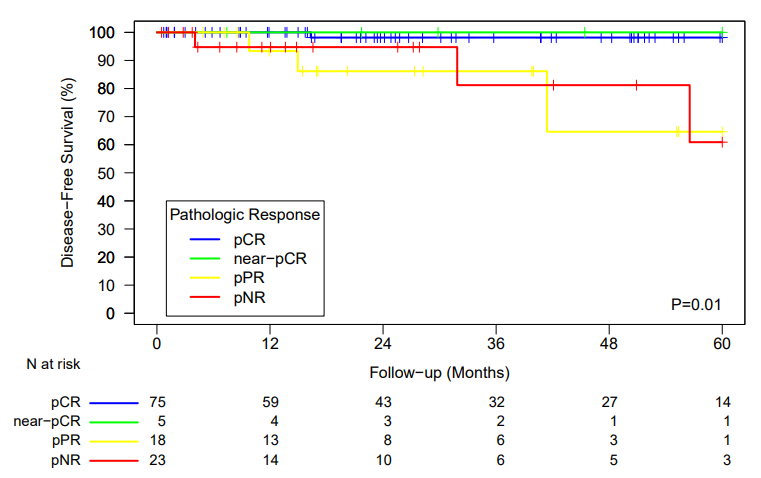

Supplement: Supplementary file 1 — Supplementary file1 (DOCX 185 kb) Fig. S1 Selection diagram for derivation of study cohort. Fig. S2 Overall survival by neoadjuvant immunotherapy regimen. Fig. S3 Overall survival by pathologic response of patients receiving neoadjuvant immunotherapy [file 10434_2026_19130_MOESM1_ESM.docx]
